# Creation of New Antimicrobial Peptides

**DOI:** 10.3390/ijms24119451

**Published:** 2023-05-29

**Authors:** Oxana V. Galzitskaya

**Affiliations:** 1 Institute of Protein Research, Russian Academy of Sciences, 142290 Pushchino, Russia; ogalzit@vega.protres.ru; 2 Institute of Theoretical and Experimental Biophysics, Russian Academy of Sciences, 142290 Pushchino, Russia

Antimicrobial peptides (AMPs) are natural compounds that exhibit potent antimicrobial activity against various microorganisms, including bacteria, fungi, and viruses. However, the use of natural AMPs in clinical settings is limited due to their stability, selectivity, and toxicity profiles. As a result, researchers are actively developing new AMPs with improved properties. Creating new AMPs involves modifying the natural peptides or designing entirely new ones with specific structural features and mechanisms of action. This approach has the potential to overcome the limitations of natural AMPs and to create peptides that are optimized for use as therapeutic agents. One of the primary benefits of creating new AMPs is the potential to overcome antibiotic resistance. Antibiotic resistance is a significant public health threat, and developing new antimicrobial agents is critical for addressing this issue. New AMPs can be designed to target specific microbial pathogens or cancer cells and have novel mechanisms of action, which could reduce the likelihood of resistance. Creating new AMPs also provides an opportunity to improve the stability and selectivity of these compounds. Natural AMPs are often susceptible to degradation by proteases, which can limit their efficacy. By modifying the structure of AMPs or incorporating new components, researchers can create peptides that are more stable and resistant to degradation. Additionally, new AMPs can be designed to improve selectivity, which could reduce the risk of harming host cells and microbiota.

Antimicrobial peptides are small molecules that play a crucial role in the innate immune system of many living organisms, including humans. However, their use as a therapeutic agent is still limited due to a lack of understanding of the relationship between the structure and activity of AMPs. In a recent study conducted by Lo et al. [1], researchers analyzed over 3000 AMPs to identify correlations between their structural properties and antimicrobial activity. Such knowledge is crucial for the rational design of novel AMPs with increased efficacy and reduced toxicity, which could contribute to developing alternative strategies and combat the growing problem of antibiotic resistance.

An important step in the creation of new AMPs is the prediction of the antimicrobial properties of selected sequences. In this regard, new tools for predicting the antimicrobial properties of peptides are being created. Burdukiewicz et al. [2] recently developed the n-gram-based AmpGram tool, which is available as an R package and web server (http://biongram.biotech.uni.wroc.pl/AmpGram/ (accessed on 3 May 2023)). As the authors note, the tool is well-suited for proteomic screening and the prediction of longer AMPs.

The prediction and search for new AMPs based on the protein sequences of the microbiome of various organisms is also a rational and fast approach at the preliminary stages of developing antimicrobial peptides. Grafskaia et al. [3] demonstrated how using computer analysis of the protein sequences of the *H. medicalis* metagenome means it is possible to identify AMP with pronounced antimicrobial activity—pept_1545—which at the same time has low toxicity to human erythrocytes. At the same time, the authors noted that the high antimicrobial effect of AMP candidates was often combined with high toxicity to eukaryotic cells. 

While AMPs have significant potential as novel antibiotics, their use has been limited by concerns about selectivity and stability under various conditions. However, recent work has shown that these issues can be overcome through modifications to known AMPs [4]. The novel antimicrobial peptide RiLK1 was highly effective against both tested Gram-negative and Gram-positive bacteria as well as fungi, and in addition, RiLK1 was structurally stable under a variety of conditions, including changes to the pH, salt and temperature conditions. Moreover, preliminary tests suggest that RiLK1 could be used to extend the shelf life of food products. These findings highlight the importance of continued research into AMPs and their potential applications in food safety.

Recently, the antimicrobial activity of cyclic lipopeptides has been studied in comparison with N-palmitoylated linear analogs [5]. Disulfide-cyclized peptides (for example, C_16_-CKRKKC-NH_2_) have shown high activity against biofilms of Candida strains, which opens prospects for the use of such antimicrobial peptides in polymers and biomaterials in medicine.

AMPs are known for their broad-spectrum activity against a variety of pathogens, including bacteria, fungi, viruses, and even cancer cells. In recent years, many research efforts have been directed toward the design and development of synthetic AMPs with improved activity, stability, and selectivity. The selectivity in the action of antimicrobial peptides (AMPs) is a critical factor in their potential use as therapeutic agents. Selectivity refers to the ability of the peptide to target microbial or cancer cells while leaving host cells intact.

Makowski et al. studied the antimicrobial activity and membrane selectivity of the EcDBS1R4 peptide [6]. These researchers also suggested that the mechanism of action for EcDBS1R4 can be related to a large lipid spatial reorganization that takes place at the inner membrane level, further highlighting the importance of understanding AMPs’ mechanisms of action when developing more effective antimicrobial therapies.

The work by Silva et al. [7] is an example of such efforts, where peptides were designed based on the conjugation of a model amphipathic cell-penetrating peptide (CPP) with tacrine. The synthesized peptides exhibited antiproliferative activity against the tumor cell lines MCF-7 and SH-SY5Y. This highlighted the potential of AMPs as a promising approach to the development of drugs against cancer. However, the development of effective AMPs is a complex task that requires significant resources, and these challenges are not limited to the design of peptides that are effective against cancer cells. 

In another study, Shagaghi et al. tested the biological activity of various analogs of the antimicrobial peptide PuroA [8]. It has been demonstrated that individual analogs (e.g., P1) have a higher antimicrobial effect compared to the original peptide. Additionally, some of the analogs showed increased resistance to proteolytic degradation, and some (P1 and Di-PuroA) even showed selectivity for cancer cells.

In another study, a 10-mer peptide was synthesized and showed selectivity for Gram-negative bacteria [9]. As the authors note, the increased permeabilization of the membrane of the Gram-negative bacteria was achieved due to the addition of Arg to the N-terminus of the original peptide, as well as the change in D-enantiomeric.

The development of synthetic antimicrobial peptides based on the rational design of an amino acid sequence made it possible to overcome several common shortcomings of natural AMPs—toxicity to mammalian cells, low selectivity, and low storage stability. Thus, Hansen et al. developed a new antimicrobial synthetic peptide based on a comprehensive analysis of natural turgencin A and optimized its shortened version [10]. As a result, the synthesized antimicrobial peptide StAMP-9, consisting of 10 natural amino acids, had a high selectivity towards pathogens and, accordingly, a low cytotoxic and hemolytic effect.

The authors developed a synthetic peptide, intestinalin, with high antistaphylococcal activity. It is noteworthy that intestinalin was created on the basis of the N-terminal region of autolysin LysC: an enzyme that is similar to bacteriophage endolysins [11]. Bacteriophages, in turn, as well as antimicrobial peptides, are one of the promising ways to fight against pathogenic bacteria, and bacteriophage lysing enzymes are a valuable resource for the development of antimicrobial agents [12].

The development of biomaterials resistant to bacterial attack is a major challenge in the field of medical applications. The authors used the cationic AMP protamine for immobilization on hydroxyapatite to block the attachment and inhibit the growth of microorganisms [13]. It can be assumed that the resulting biomaterial with antibacterial properties could be further used, for example, in bone tissue engineering or for the support of bone tissue regeneration.

Creating hybrid AMPs is an emerging strategy with significant potential for improving the effectiveness of antimicrobial therapies. Hybrid AMPs can combine the beneficial properties of natural AMPs with synthetic components to create peptides with enhanced activity, stability, selectivity, and resistance. This approach has the potential to lead to more effective and targeted therapies for infectious diseases.

The combination of two fragments of peptides, each of which has antimicrobial properties, was used in the work of Yang et al. [14]. The new hybrid peptide CA-FO containing the N-terminal fragment of cecropin A (CA (1–8) and a fragment of fowlicidin-2 (1–15) had high antimicrobial activity.

Programs for predicting amyloidogenic regions have been used to select amyloidogenic regions for future antimicrobial peptides [15]. In addition, antimicrobial peptides containing a CPP fragment at the N-terminus and an amyloidogenic fragment of the S1 ribosomal protein at the C-terminus were developed and synthesized [16]. The obtained hybrid peptides showed high potential as antimicrobial agents. The mechanism of action of such amyloidogenic peptides with antimicrobial activity was also proposed, which is based on the directed aggregation of the peptide with the functionally important S1 ribosomal protein. The authors synthesized and tested the properties of peptides based on the predicted amyloidogenic regions of the S1 ribosomal protein. Similarly, hybrid amyloidogenic-antimicrobial peptides were designed to target coaggregate with the parent functionally important protein to turn off its function in the pathogenic microorganism [17].

In addition, some proline-rich antimicrobial peptides (PrAMPs) could be of interest in the treatment of infections caused by ESKAPE pathogens. Thus, it was recently shown that two PrAMPs of five cetacean species (cePrAMPs) exhibited a broad spectrum of antibacterial activity at a minimum inhibitory concentration (MIC) ≤ 4 µM [18].

Previously, 201 genomes of *Pseudomonas aeruginosa*, *Staphylococcus aureus*, *Enterobacter* and *Mycoplasma* were analyzed, and annotations of KEGG pathways were used to reveal the characteristic metabolic features of these pathogens [19]. The information obtained in this way can be used to identify the main pathways of pathogen resistance to antibiotics.

The high resistance of microorganisms to traditional antibiotics is a growing public health problem. At the same time, this task stimulates the development of new strategies for the search and creation of effective antimicrobial agents. Some harmful side effects can be avoided, for example, through peptide sequence mimetic strategies [20]. Antimicrobial peptides can exhibit high antimicrobial activity, but they also need some improvements, particularly in structure optimization, to reduce toxicity to human cells and increase stability under physiological conditions and under the storage conditions of preparations.

The practical use of AMPs can be expanded by increasing their bioavailability, specificity, and activity through lipidization, i.e., adding one or more fatty acid chains to lysine residues or the N-terminus of the peptide [21]. In combination with other approaches, such as the cyclization of the peptide, and the use of non-natural amino acids, it is hoped that an effective AMP meeting the given requirements can be created. Such lipids could be targeted to a particular type of membrane or receptor.

Antimicrobial peptides containing amyloidogenic regions corresponding to the same amyloidogenic fragments in the functionally important proteins of microorganisms can be effective not only against bacteria but also against viruses [17]. It is known that the amyloidogenic properties of S-proteins in different strains of coronaviruses can differ significantly [22]. At the same time, it was assumed that there was some connection between the high amyloidogenicity of the S-protein and the high infectivity of the coronavirus strain. AMPs can act through various mechanisms, including the directed coaggregation mechanism. In order to develop new antiviral peptides, it is possible to use the amyloidogenic regions of individual viral proteins as fragments of new AMPs that act through coaggregation with viral proteins that block the attachment and spread of the virus.

De novo developed β-boomerang and α-helical AMPs were discussed in a recent review [23]. The authors illustrated that the function of the AMP is related to its structure. At the same time, rational design, chemical modifications, and the optimization of the delivery of antimicrobial peptides provide a high antimicrobial effect.

AMPs can be effective against pathogenic bacteria if they quickly destroy the bacterial membrane, do not lead to the development of multidrug resistance, and are safe for humans. Currently, there are still a few AMPs that have passed clinical trials and exist as commercially available antimicrobials. At the same time, in the long term, it is important not to make the same mistakes that have arisen for traditional antibiotics, including excessive and uncontrolled use [24].

**Conclusions**: One of the major benefits of creating new AMPs is the potential to overcome resistance. Antibiotic resistance is a significant public health threat, and the development of new antimicrobial agents is critical for addressing this issue. New AMPs can be designed to target specific microbial pathogens and to have novel mechanisms of action, which could reduce the likelihood of resistance. In conclusion, the creation of new antimicrobial peptides is a critical area of research with significant potential for addressing the problem of antibiotic resistance. New AMPs can overcome the limitations of natural peptides and provide opportunities to develop more targeted and effective therapies for infectious and cancer diseases. However, further research is needed to optimize the properties of new AMPs and to evaluate their safety and efficacy in clinical settings.
